# Evaluation of omadacycline *in vitro* activity against *Corynebacterium* species

**DOI:** 10.1128/aac.01864-24

**Published:** 2025-03-12

**Authors:** Saltanat Ualiyeva, Justin McCallum, Alexander Donald Pyden, Zoe Freeman Weiss

**Affiliations:** 1 Department of Pathology and Laboratory Medicine, Tufts Medical Center 1867 , Boston, Massachusetts, USA; 2 Department of Pathology and Laboratory Medicine, Lahey Hospital & Medical Center 2094 , Burlington, Massachusetts, USA; 3 Division of Geographic Medicine and Infectious Diseases, Tufts Medical Center 1867 , Boston, Massachusetts, USA; The Peter Doherty Institute for Infection and Immunity, Melbourne, Victoria, Australia

**Keywords:** susceptibility, omadacycline, *Corynebacterium*

## Abstract

*Corynebacterium* spp. are gram-positive bacteria increasingly recognized as pathogens. This study evaluates the MICs of omadacycline, a tetracycline, against 40 clinical *Corynebacterium* isolates using two methodologies: broth microdilution (BMD) and Liofilchem omadacycline MIC Test Strip (MTS). By BMD, the MIC_50_, MIC_90_, and MIC range were 0.5 µg/mL, 1 µg/mL, and 0.12–2.0 µg/mL, respectively. Comparing BMD to MTS, essential agreement (EA, within ±1 doubling dilution of the reference BMD MIC) was 87.5% (95% CI: 73.0%–95.4%).

## INTRODUCTION

*Corynebacterium* species are gram-positive, non-spore-forming, rod-shaped bacteria. Most of the *Corynebacterium* spp. isolated from clinical samples (C. *striatum*, *C. aurimucosum*, *C. amycolatum*, *C. macginleyi*) represent non-pathogenic skin flora but are occasionally capable of becoming true pathogens (1–4) especially in immunocompromised individuals, wound infections (2–4), and ventilator-associated pneumonia (5). Other species such as *C. jeikeium* (6, 7) and *C. urealyticum* (8) are associated with opportunistic infections and urologic infections, respectively. *Corynebacterium* spp. have, in recent years, been implicated in chronic left ventricular assist device (LVAD) driveline infections (9, 10), often requiring chronic long-term antibiotic therapy. Oral antibiotics are often preferred over intravenous antibiotics for convenience of use, reduced healthcare costs, and decreased risk of infections. Tetracycline antibiotics are attractive oral options for the treatment of coryneform organisms given their limited side effect profile and gram-positive coverage; however, many *Corynebacterium* spp. are resistant to tetracyclines including doxycycline (11, 12).

Omadacycline is a novel oral and intravenous antibiotic, which inhibits bacterial protein synthesis by binding to the 30S ribosomal subunit similar to other tetracyclines (13, 14). There is limited data on the *in vitro* activity of omadacycline against *Corynebacterium* spp. In addition, no clinical studies have been performed, and omadacycline is not currently clinically indicated for the treatment of infections due to *Corynebacterium* spp. as such there are no established susceptibility interpretive criteria (breakpoints). Given the extent of resistance against other tetracycline class drugs (up to 75% of isolates in some data sets), evaluating the activity of omadacycline, may increase the number of oral options (15, 16). Omadacycline evades common tetracycline class resistance mechanisms, efflux, and ribosomal protection and, therefore, may retain activity against tetracycline-resistant *Corynebacterium* spp. (13). Broth microdilution (BMD) is considered the gold standard for antibiotic susceptibility testing (AST) but can be resource-intensive and requires specialized training and materials, making it less feasible for routine use in many clinical laboratories. Pre-defined panels are available on automated instruments which are faster than manual BMD; however, newer antibiotics may not be integrated into these panels for years. Laboratories must rely on manual methods, such as minimum inhibitory concentration (MIC) Test Strip [MTS] or disk diffusion methods when testing newer antibiotics locally (17). Disk diffusion testing of *Corynebacterium* spp. is not currently recommended by the Clinical and Laboratory Standards Institute (CLSI) (18). While E-tests are available for use against *Corynebacterium* spp., usage is on a research use only basis (19), and variable performance in the literature has limited their widespread use. While several studies have demonstrated good performance of MTS, specifically E-tests, compared to reference methods (20–22), there are some concerns regarding the performance of MTS for specific drugs. One study comparing MTS to BMD demonstrated antibiotic-specific variability in overall essential agreement (EA), concordance within ±1 doubling dilution of the reference BMD MIC, between these methods in coryneform bacteria (ampicillin, EA = 84%; cephalothin, 88%; cefoxitin, 77%; cefotaxime, 71%; erythromycin, 87%; ciprofloxacin, 77%; tetracycline, 79%; amikacin, 64%; vancomycin, 31%; and rifampin, 88%) (23).

A study across multiple institutions compared the *in vitro* performance of omadacycline MTS (Liofilchem MIC Test Strips [MTS], Roseto degli Abruzzi, Italy) to BMD against gram-negative, gram-positive, and fastidious bacteria and showed agreement of above 90% of the MIC values obtained against the majority of isolates (24).

A recent surveillance study performed by JMI Laboratories demonstrated *in vitro* activity of omadacycline and comparator agents against a number of pathogens, including *Corynebacterium* spp. (25). This study tested 10 *Corynebacterium* isolates, including *C. amycolatum* (1), *C. jeikeium* (1), *C. simulans* (1), *C. striatum* (6), and *C. urealyticum* (1), and their susceptibility against omadacycline, doxycycline, and erythromycin. Omadacycline had the lowest MIC_50/90_ (0.12/0.5 µg/mL) compared to doxycycline (0.25/8 µg/mL) and erythromycin (8/8 µg/ml) (24). One published study reports a case of polymicrobial infection including *Acinetobacter baumanni*, *coagulase negative Staphylococcus,* and *Corynebacterium* that was successfully treated with omadacycline (26).

The primary objective of our study was to evaluate the *in vitro* activity of omadacycline against clinically significant *Corynebacterium* spp. isolates collected at our institution. A secondary objective was to compare the performance of MTS with the standard BMD method.

A total of 40 clinical isolates identified by mass spectrometry were collected at our institution from 2022 to 2024. Isolates were included only if clinicians had requested susceptibility testing be performed, indicating a higher probability of clinical relevance (full AST was not ultimately performed in all cases). The majority of our clinical isolates were wound cultures (*N* = 28) from patients who have an LVAD or other indwelling devices, followed by blood (*N* = 5), corneal (*N* = 5), and respiratory cultures (*N* = 2). Some isolates were referred for routine susceptibility panels, of which 100% (*N* = 26) were susceptible (S) to vancomycin, linezolid, and daptomycin. Susceptibility was lower to oral agents: ciprofloxacin (7/25, 28%S), doxycycline (12/14, 86%S), tetracycline (10/25, 40%S), penicillin (3/26, 12%S), clindamycin (3/25, 12%S).

**Fig 1:**
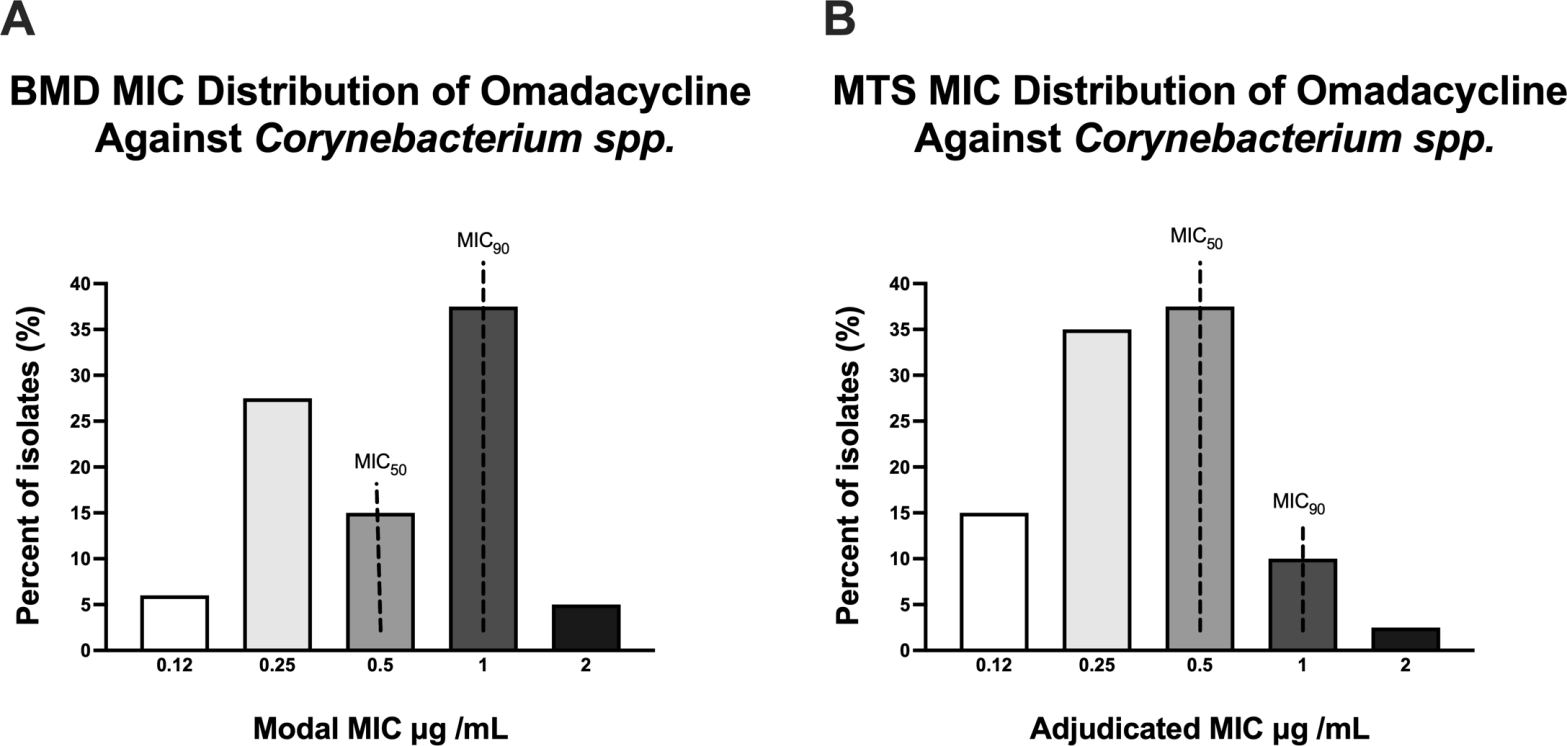
Distribution of the modal minimum inhibitory concentration (MIC) for omadacycline by reference broth microdilution (BMD) (**A**) and adjudicated MIC distribution by MTS (Liofilchem). (**B**) Adjudicated MIC represents modal MIC, which is the most frequent MIC value observed. Median used in cases without a modal MIC.

Isolates were sent out to a reference laboratory (Laboratory Specialists Inc., OH) for MIC testing by BMD, performed in triplicate using CLSI methods (27). The isolates were tested in frozen panels of Cation-Adjusted Mueller Hinton Broth supplemented with 2.5%–5% lysed horse blood (27), and MICs were recorded. MIC distributions by BMD are shown in Fig. 1. The MIC_50_, MIC_90_, and MIC range were calculated using standard CLSI methods (27).

MTS testing was performed at our institution in triplicate following CLSI guidelines (CLSI M07) (27). A standardized bacterial suspension (0.5 McFarland) was spread uniformly on the surface of a Blood Mueller-Hinton agar plate (Becton Dickinson) and incubated with Liofilchem MTS (Liofilchem, Roseto degli Abruzzi, Italy). Replicates for each isolate were performed by different technicians. The MIC was defined as the point where the elliptical zone of inhibition intersected the MTS, as per manufacturer instructions. *Streptococcus pneumoniae* ATCC 49619 strain was used for quality control in both BMD and MTS.

EA defined as concordance within ±1 twofold dilution of the reference BMD MIC was reported (28). Confidence intervals were calculated using the Wilson Score Interval (29).

The MIC_50_, MIC_90_, and MIC range are reported in Table 1. Compared to MTS, EA was 87.5% (95% CI: 0.730, 0.954); however, results were not statistically significant given the sample size. These findings are similar to previous literature demonstrating <90% EA between BMD and MTS for *Corynebacterium* spp. against most antibiotics. The reason for these discrepancies is not entirely clear. The susceptible breakpoint for omadacycline against other gram positives ranges from <= 0.12 µg/mL to <= 0.5 µg/mL. Low MIC_50_ values against *Corynebacterium* spp. suggest that omadacycline could be a potentially active oral treatment option. Further data are required to determine whether MICs in this range correlate to clinical success against *Corynebacterium* spp. This small single-center study has a limited sample size, which prevents us from drawing definitive conclusions about the suitability of MTS for omadacycline AST and precludes species-specific analysis.

**TABLE 1:** Results of MIC testing by BMD and MTS for omadacycline^*a*^

Isolate number	Organism name	BMD			MTS			BMD modal MIC, µg/mL	Adjudicated MIC MTS^*b*^, µg/mL	EA
		Replicate 1	Replicate 2	Replicate 3	Replicate 1	Replicate 2	Replicate 3			
1	*C. accolens*	0.25	0.25	0.25	0.5	0.5	0.5	0.25	0.5	yes
2	*C. amycolatum*	1	1	1	2	1.5	1.5	1	2	yes
3	*C. amycolatum*	0.25	0.25	0.25	0.5	0.25	0.5	0.25	0.5	yes
4	*C. amycolatum*	1	2	2	2	1	1	2	1	yes
5	*C. aurimucosum*	0.25	0.25	0.25	0.12	0.5	0.25	0.25	0.25	yes
6	*C. aurimucosum*	0.25	0.25	0.25	0.25	0.25	0.25	0.25	0.25	yes
7	*C. auriscanis*	0.5	0.5	0.5	0.25	0.125	0.25	0.5	0.25	yes
8	*C. macginleyi*	0.12	0.25	0.25	0.5	0.25	0.25	0.25	0.25	yes
9	*C. macginleyi*	0.12	0.12	0.12	0.25	0.25	0.25	0.12	0.25	yes
10	*C. minutissimum*	0.25	0.25	0.25	0.125	0.125	0.125	0.25	0.125	yes
11	*C. propinquum*	1	1	1	0.5	0.5	0.5	1	0.5	yes
12	*C. striatum*	0.5	0.5	0.5	0.25	0.25	0.125	0.5	0.25	yes
13	*C. striatum*	2	2	2	0.25	2	1	2	2	yes
14	*C. striatum*	1	1	1	0.25	0.25	0.25	1	0.25	no
15	*C. striatum*	1	1	1	0.5	0.5	0.5	1	0.5	yes
16	*C. striatum*	1	1	1	0.25	0.5	0.5	1	0.5	yes
17	*C. striatum*	0.12	0.12	0.12	0.12	0.12	0.12	0.12	0.12	yes
18	*C. striatum*	1	1	1	0.25	0.5	0.5	1	0.5	yes
19	*C. striatum*	0.12	0.12	0.12	0.125	0.125	0.125	0.12	0.125	yes
20	*C. striatum*	0.5	1	0.5	0.25	0.25	0.25	0.5	0.25	yes
21	*C. striatum*	0.12	0.12	0.12	0.5	1	0.25	0.12	0.5	no
22	*C. striatum*	1	1	1	0.25	0.25	0.25	1	0.25	no
23	*C. striatum*	0.25	0.25	0.25	0.25	0.5	0.5	0.25	0.5	yes
24	*C. striatum*	1	1	1	1	1	1	1	1	yes
25	*C. striatum*	1	1	1	0.5	0.5	0.25	1	0.5	yes
26	*C. striatum*	1	1	1	0.5	0.5	0.5	1	0.5	yes
27	*C. striatum*	0.25	0.25	0.25	0.25	0.25	0.5	0.25	0.25	yes
28	*C. striatum*	1	1	1	1	1	1	1	1	yes
29	*C. striatum*	0.25	0.25	0.25	0.5	0.25	0.25	0.25	0.25	yes
30	*C. striatum*	1	1	1	0.25	0.5	0.5	1	0.5	yes
31	*C. striatum*	0.25	0.25	0.25	0.5	0.25	0.25	0.25	0.25	yes
32	*C. striatum*	1	1	1	0.5	1	0.5	1	0.5	yes
33	*C. striatum*	1	1	1	0.25	0.25	0.5	1	0.25	no
34	*C. striatum*	0.12	0.12	0.12	0.12	0.25	0.012	0.12	0.12	yes
35	*C. striatum*	0.25	0.25	0.25	0.25	0.5	0.12	0.25	0.25	yes
36	*C. striatum*	0.12	0.12	0.12	0.12	0.12	0.12	0.12	0.12	yes
37	*C. striatum*	0.5	0.5	0.5	0.5	0.5	0.5	0.5	0.5	yes
38	*C. tuberculostearicum*	0.5	0.5	0.5	0.5	0.5	0.5	0.5	0.5	yes
39	*C. urealyticum*	0.5	0.5	0.5	0.25	0.5	1	0.5	0.5	yes
40	*C. urealyticum*	1	1	1	0.12	0.06	0.25	1	0.12	no
						MIC_50_ (µg/mL)		0.5	0.5	
						MIC_90_ (µg/mL)		1	1	
						MIC range (µg/mL)		0.12–2.0	0.12–2.0	
									EA	87.5% (95% CI: 73.0–95.4%)

^*a*^ Species represented include *Corynebacterium striatum* (*N* = 26), *C. urealyticum* (*N* = 2), *C. amycolatum* (*N* = 3), *C. minutissimum* (*N* = 1), C. *macginleyi* (*N* = 2), *C. accolens* (*N* = 1), *C. propinquum* (*N* = 1), *C. auriscanis* (*N* = 1), *C. tuberculostearicum* (*N* = 1), and *C. aurimucosum* (*N* = 2).

^*b*^ Adjudicated MIC represents modal MIC. Median used in cases without a modal MIC.

To date, this is the largest study demonstrating *in vitro* activity of omadacycline against *Corynebacterium* spp.; however, our study did not demonstrate >90% EA when comparing BMD to MTS. Further research with larger multicenter data sets, diverse geographic representation, and clinical outcome data is necessary to determine the clinical and microbiological relevance of omadacycline AST against *Corynebacterium* spp.
